# Comment on "Evaluation of aortic stiffness with an oscillometric arteriograph device in patients with hypertension and ascending aortic aneurysm"

**DOI:** 10.1590/1806-9282.20252275

**Published:** 2026-06-19

**Authors:** Huseyin Durmaz, Semiha Durmaz

**Affiliations:** 1 Konya City Hospital, Department of Cardiovascular Surgery – Konya, Turkiye.; 2 Konya City Hospital, Department of Internal Medicine – Konya, Turkiye.

Dear Editor,

I have read with interest the article entitled "Evaluation of aortic stiffness with an oscillometric arteriograph device in patients with hypertension and ascending aortic aneurysm," recently published in the journal Revista da Associação Médica Brasileira by Dr. Arpa et al.^1^. The study is an important and timely investigation as it highlights the potential relationship between the development of ascending aortic aneurysms and increased aortic stiffness.

The data presented in the study clearly reveal the mechanism of ascending aortic aneurysm development. In this sense, the recommendations presented for utilizing the augmentation index (Aix) and pulse wave velocity (PWV), two parameters of critical importance in determining arterial stiffness, for the measurement of aortic stiffness are quite important. However, I believe there are some points that need to be addressed in this study.

PWV is not the only measure that is important for arterial stiffness in the presence of hypertension and multiple risk factors. The study's limitations include mentioning genetic predispositions and lifestyle factors that can affect aortic stiffness and aneurysm progression. However, arterial stiffness increases in diabetes and chronic inflammation^2^. Diabetes and chronic inflammation were not mentioned in the study.

PWV is a measurable parameter. The total (intimal-medial-adventitial) wall thickness carrying the PWV load is related to the bulk density of blood and the lumen radius^3^. The bulk density of blood is one of the determinants of blood viscosity. In addition, blood viscosity is determined by body temperature, hematocrit, erythrocyte deformability, and especially the plasma concentrations of some components such as fibrinogen, low-density lipoprotein, high-density lipoprotein, and albumin^4^. Although the study mentioned the determinants of blood viscosity, it did not mention determinants such as body temperature, erythrocyte deformability, and albumin. I believe that mentioning other parameters affecting blood viscosity in the study would contribute to a better understanding of the PWV parameter, which is critical for measuring aortic stiffness.

In conclusion, Dr. Arpa et al.'s study provides valuable information by addressing the relationship between the development of ascending aortic aneurysms and aortic stiffness. I eagerly await further research in this area and hope that the authors will consider these suggestions in their future studies.

Thank you for giving me the opportunity to provide feedback on this important study.

Yours truly,

## Data Availability

The datasets generated and/or analyzed during the current study are available from the corresponding author upon reasonable request.

## References

[1] Arpa A, Özbek M, Güzel T, Polat N (2026). Evaluation of aortic stiffness with an oscillometric arteriograph device in patients with hypertension and ascending aortic aneurysm. Rev Assoc Med Bras.

[2] Wagenseil JE, Mecham RP (2009). Vascular extracellular matrix and arterial mechanics. Physiol Rev.

[3] Boutouyrie P, Chowienczyk P, Humphrey JD, Mitchell GF (2021). Arterial stiffness and cardiovascular risk in hypertension. Circ Res.

[4] Sloop GD, Pop G, Weidman JJ, St Cyr JA (2024). Hormonal control of blood viscosity. Cureus.

